# Risk of complications in vascular surgery: development of a clinical predictive model

**DOI:** 10.1590/1677-5449.202501812

**Published:** 2026-04-10

**Authors:** Juliana Peres, Jeferson Freitas Toregeani, Ana Julia Vendrametto

**Affiliations:** 1 Centro Universitário Fundação Assis Gurgacz – FAG, Cascavel, PR, Brasil.; 2 Universidade Estadual do Oeste do Paraná – UNIOESTE, Cascavel, PR, Brasil.

**Keywords:** vascular surgery, postoperative complications, risk assessment

## Abstract

**Background:**

Postoperative complications in vascular surgery are associated with high morbidity, mortality, and hospital costs, highlighting the need for reliable predictive tools for risk stratification.

**Objectives:**

To develop and validate a clinical model to estimate the risk of postoperative complications in vascular surgery.

**Methods:**

This retrospective study included 510 patients who underwent vascular surgeries between 2021 and 2024, divided into arterial, venous, and vascular access subgroups. Clinical and surgical variables were analyzed using multivariate logistic regression, and model performance was evaluated using the receiver operating characteristic curve.

**Results:**

The overall complication rate was 17.6%, being higher in arterial procedures (35.6%) than venous procedures (11.3%) or vascular access surgeries (6.9%). In the total sample, age (odds ratio [OR] 1.03; p = 0.006), chronic kidney disease (OR 9.94; p < 0.001), smoking (OR 3.29; p = 0.001), and procedure time (p = 0.038) were independent predictors, while chronic anticoagulant use had a protective effect (OR 0.39; p = 0.036). In the specific subgroup models, type 2 diabetes mellitus (OR 13.54; p < 0.001) and chronic kidney disease (OR 15.30; p = 0.007) were significant predictors in the venous access group, smoking was associated with risk in the vascular access group (OR 9.57; p = 0.081), and chronic kidney disease was significant in the arterial group (OR 6.50; p < 0.001). The model showed good discriminatory performance (overall area under the curve [AUC] = 0.806).

**Conclusions:**

The proposed model demonstrated good accuracy and clinical applicability, allowing individualized risk stratification across different vascular surgery contexts. External validation is needed to confirm its usefulness.

## INTRODUCTION

Vascular surgery, an essential and constantly evolving field of medicine, addresses a wide range of diseases affecting the circulatory system, from peripheral arterial occlusive disease to the repair of complex aneurysms and the revascularization of vital areas such as the carotid arteries.^1,2^ In recent decades, advances in surgical techniques, both open and endovascular, and improvements in perioperative management have enabled the treatment of increasingly complex patients with multiple comorbidities.^3,4^ However, despite these advances, postoperative complications remain a significant challenge, with a profound impact on patient morbidity, mortality, and quality of life.^5-7^

Multicenter studies and large-scale registries, such as the UK’s National Vascular Registry, demonstrate that the incidence of complications in vascular surgery remains high, with rates ranging from 15% to 30%, depending on the type of procedure, the urgency of the surgery, and the patient’s baseline clinical conditions.^8,9^ Complications such as surgical site infection, major cardiovascular events (acute myocardial infarction or stroke), acute renal failure, respiratory complications, and the need for reintervention are frequently reported, even by centers of excellence.^10-14^ In particular, myocardial injury following non-cardiac surgery has been identified as a common complication and is strongly associated with increased short- and long-term mortality.^7,15^

The profile of patients who undergo vascular surgery is characterized by a high prevalence of cardiovascular risk factors, such as advanced age, diabetes mellitus, systemic arterial hypertension, chronic kidney disease (CKD), and smoking.^13,16^ These comorbidities not only increase the complexity of clinical management, but are also independent predictors of adverse outcomes.^17,18^ The effects of these pre-existing conditions on prognosis is widely documented; studies have shown that patients with multiple comorbidities have a higher risk of infectious, respiratory, and cardiovascular complications, in addition to longer hospital stays and associated costs.^19,20^

In this scenario, individualized risk stratification becomes a fundamental tool for clinical decision-making, surgical planning, and optimization of perioperative care. ^12^ Several risk scores have been developed and applied in general and cardiac surgery, such as the Revised Cardiac Risk Index^21^ and the American Society of Anesthesiologists score.^22^ However, the applicability and accuracy of these models in cohorts of vascular patients are often limited, and they may underestimate the risk in highly complex populations.^23-26^.

In response to this gap, specific tools for vascular surgery have been proposed, such as the Vascular Study Group of New England Cardiac Risk Index^27^ and the New Zealand Vascular Surgical Risk Tool,^28^ which have demonstrated better performance in specific populations.^29,30^ More recently, models based on machine learning and artificial intelligence have emerged as promising alternatives, offering dynamic and personalized prediction based on large volumes of data.^31,32^ However, many of these tools still lack broad external validation or are not readily accessible in daily clinical practice.

Therefore, the development of a risk calculator that is practical, accessible, validated, and specifically designed for the population of patients undergoing vascular surgery in our setting remains a pressing need. The objective of this study was to develop a lightweight tool based on independent predictors of postoperative complications that is clinically and immediately applicable. Such an initiative could help reduce complications and improve quality of care and patient safety in the context of vascular surgery.^28,33,34^

## METHODS

### Study type and location

This retrospective observational study was approved by the Human Research Ethics Committee of the Fundação Assis Gurgacz University Center (opinion 7.149.647, certificate 83271224.7.0000.5219). Conducted over 1 year, it aimed to analyze the medical records of patients undergoing vascular surgery at a hospital in the western region of Paraná, Brazil. Considering the study’s retrospective nature and the large population involved, the requirement for informed consent was waived by the ethics committee.

### Study population

We used a consecutive selection method, including all patients who underwent vascular surgery procedures between 2021 and 2024 at a teaching hospital in Western Paraná. Patients < 18 years of age and those lacking essential pre- or post-operative data for determining the outcome, defined as the occurrence of a post-operative complication, were excluded from the study.

**Figure 1:**
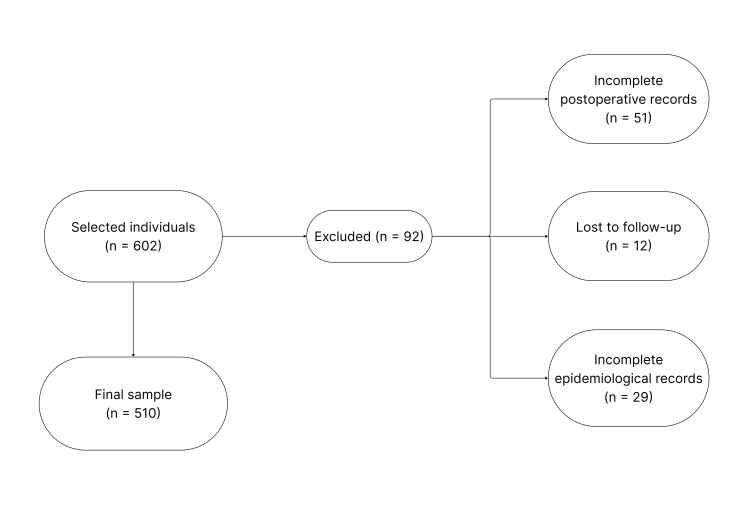
Patient selection flowchart.

To minimize selection risk, the inclusion and exclusion criteria were standardized (see Figure 1), ensuring sample representativeness without subjective interference. Exclusions were limited to cases that could compromise the complication analysis and, thus, distort the results. Multivariate logistic regression analysis was used to control for confounding factors, making the evaluation more precise and reliable.

In total, 602 individuals were selected, of whom 92 were excluded. Of these, 51 did not have sufficient postoperative records to indicate whether there were complications, 12 were lost to clinical follow-up after surgery at the hospital, and 29 had incomplete epidemiological records.

To ensure consistent statistical power, a minimum sample size of 142 participants was calculated, based on a formula for finite populations presented below in Equation 1, considering an incidence of post-surgical complications = 15%,^8,9,11,21^ n = 510, 95% CI, error = 5%. However, it was decided to include all 510 eligible cases to increase the precision of the estimates.

Equation 1:


$$\text{n =}\left\{\begin{array}{c} \left[\text{510 x 0.15 x}\left(\text{1 - 0.15}\right)\text{x}\left(\text{1.96}\right)\text{²}\right]\text{/}\\ \left[\left(\text{510 - 1}\right)\text{x}\left(\text{0.05}\right)\text{²}\right]\text{+}\\ \left[\text{0.15 x}\left(\text{1 - 0.15}\right)\text{x}\left(\text{1.96}\right)\text{²}\right] \end{array}\right\}$$ (1)

### Study variables

The variables collected through electronic medical records were: sex, age, weight, race, pre-existing comorbidities such as systemic arterial hypertension, type 2 diabetes mellitus (DM2), chronic artery disease, CKD, dyslipidemia, and history of thrombosis and smoking, as well as the type of vascular procedure, procedure time, anesthesia type, and the occurrence of postoperative complications.

### Main outcome

The primary outcome was the occurrence of postoperative complications in patients undergoing vascular surgery. Complications included events such as the need for blood transfusion, surgical wound infection, surgical reintervention, thrombotic events, cardiovascular complications, and death in the immediate postoperative period. A predictive risk model was also developed, based on clinical, demographic, and surgical variables, with stratified analysis by subgroups of arterial, venous, and vascular access procedures, to allow individualized risk stratification and identification of specific factors associated with each type of surgery.

### Data analysis

The collected data, which were synthesized and organized in an Excel spreadsheet, were analyzed descriptively and inferentially. Continuous variables (age, weight, and procedure time) were presented as mean (SD) for normally distributed variables, and as median and interquartile range for non-normally distributed variables. Normality was verified using the Shapiro-Wilk test. Categorical variables were expressed as absolute and percentage frequencies. Comparisons between groups (patients with and without postoperative complications after vascular surgery) for continuous variables were performed using the Mann-Whitney test (non-parametric) in a listwise analysis, due to the non-normal distribution. Categorical variables were compared using the χ2 test or Fisher’s exact test, when appropriate. Binomial logistic regression analysis was used to identify factors associated with postoperative complications, using the backward stepwise method and the Wald statistic. Odds ratios (OR) ​​and their respective 95% CI were presented to quantify the magnitude of the associations, and p-values < 0.05 were considered statistically significant in all analyses.

Descriptive and bivariate analyses were performed in GraphPad Prism 10.3.9 and Jamovi 2.4. Multivariate logistic regression was performed in the Jamovi regression module with extraction of coefficients (β), OR, and adjustment measures.

Individual risk was calculated according to the results of binary logistic regression, where the predicted probabilities were categorized as low (< 20%), intermediate (20%-50%), or high (> 50%), which facilitates the clinical applicability of the score. Candidate variables were based on clinical relevance and statistical association in the bivariate analysis. The backward stepwise method, with Wald statistics, defined the final model. The logistic function applied the estimated coefficients to the included clinical predictors. Continuous variables were entered in their original form (age in years), and categorical variables were coded as dichotomous (0 = absent; 1 = present). The model’s calibration and discrimination were evaluated using the receiver operating characteristic curve, and the cut-off point for binary classification (high vs low risk) was defined by the Youden index^35^ (J = sensitivity + specificity - 1), which seeks the best balance between sensitivity and specificity. For presentation purposes, the predicted probabilities were also categorized into risk ranges (low, intermediate, and high), as recommended in the literature.^23^

## RESULTS

A total of 602 patients who underwent vascular procedures were evaluated, of whom 92 were excluded (51 without postoperative records, 12 due to loss to follow-up, and 29 due to incomplete data), leaving 510 patients in the analysis with complete information. Due to the clinical heterogeneity among vascular surgery procedures, the analysis was stratified into 3 subgroups: arterial and atherosclerotic diseases (n = 146), venous access (n = 292), and vascular access (n = 72). The sample was predominantly female (67.8%) and White (81.2%). Their age ranged from 18 to 94 years, with a mean of 58.8 (SD, 15.6) years and a median of 60 years. Body weight ranged from 39 to 150 kg, with a mean of 73.1 (SD, 14.2) kg and a median of 70.2 kg. The demographic and clinical characteristics of each subgroup are shown in Table 1.

**Table 1:** Demographic and clinical characteristics of a sample of 510 patients who underwent vascular surgery procedures between 2021 and 2024 at a teaching hospital in western Paraná, Brazil.

**Demographic variables**	**Venous (n = 292)**	**Vascular access (n = 72)**	**Arterial (n = 146)**
	n (%) or mean ± SD		
Age (years)	52.4±13.8	63.4±15.9	69.2±12.1
Weight (kg)	74.9±14.5	72.0±13.0	70.0±13.5
Female	232 (79.4%)	39 (54.2%)	75 (51.4%)
Male	60 (20.6%)	33 (45.8%)	71 (48.6%)
White	232 (79.5%)	66 (91.7%)	116 (79.5%)
Black	2 (0.7%)	1 (1.4%)	1 (0.7%)
Mixed race	48 (16.4%)	5 (6.9%)	28 (19.2%)
Asian	10 (3.4%)	0	1 (0.7%)
Clinical variables diagnosed prior to the vascular procedure.			
Hypertension	45 (15.4%)	22 (30.6%)	114 (78.1%)
Type 2 diabetes mellitus	22 (7.5%)	5 (6.9%)	70 (47.9%)
Chronic arterial disease	3 (1.0%)	2 (2.8%)	23 (15.8%)
Chronic kidney injury	12 (4.1%)	59 (81.9%)	22 (15.1%)
Dyslipidemia	16 (5.5%)	7 (9.7%)	78 (53.4%)
Smoking	11 (3.8%)	5 (6.9%)	48 (32.9%)
Continuous use of anticoagulants/antiplatelet agents	9 (3.1%)	0	56 (38.4%)
Patient with a history of previous thrombotic event.	9 (3.1%)	4 (5.6%)	38 (26.0%)

SD = standard deviation. Source: research data.

The most frequent procedure was bilateral varicose vein surgery (57.3%), followed by arteriovenous fistula creation (12.5%) and femorodistal revascularization (6.7%). More complex procedures, such as repair of ruptured abdominal aortic aneurysms (1.8%) and carotid endarterectomy (5.5%), also occurred, although in smaller proportions. The predominant anesthetic technique varied significantly between the groups. Spinal anesthesia combined with sedation was the most common type in the venous (n = 256; 87.7%) and arterial groups (n = 43; 29.5%), while local anesthesia combined with sedation was the most common type in the vascular access group (n = 51; 70.8%). General anesthesia was more prevalent in the arterial group (n = 95; 65.1%). Regarding procedure duration, longest mean time occurred in the arterial group, with a mean of 164 (SD, 68.5) minutes and a median of 150 minutes. Procedure times in the venous and vascular access groups were shorter, with means of 102 (SD, 41.0) minutes and 84.4 (SD, 45.2) minutes, respectively.

The postoperative complication rate was 17.6% (n = 90) in the total sample, with a large disparity between subgroups. The arterial group had the highest complication rate (n = 52; 35.6%), followed by the venous group (n = 33; 11.3%) and the vascular access group (n = 5; 6.9%). Among the specific events, the need for reoperation was more frequent in the arterial (n = 13; 8.9%) and venous (n = 14; 4.8%) groups, being rare in the vascular access group (n = 2; 2.8%). Surgical wound infection or poor healing occurred in 5.8% of the arterial group (n = 14) and 5.8% of the venous group (n = 17). Serious events, such as postoperative thrombosis (n = 11; 7.5%), intense intraoperative blood loss (n = 22; 15.1%), and the need for transfusion (n = 23; 15.8%), occurred almost exclusively in the arterial group. Acute kidney injury was also more prevalent in the arterial group (n = 19; 13.0%), and hospital deaths occurred only in this subgroup (n = 7; 4.8%).

In the bivariate analysis of clinical variables in relation to postoperative complications in the total sample, age was significantly higher among patients with complications (U = 12,513; p < 0.001), as was procedure time (U = 13,924; p < 0.001). Among categorical variables, complications were associated with hypertension (χ^2^ = 19.2; p < 0.001), DM2 (χ^2^ = 41.9; p < 0.001), CKD (χ^2^ = 16.7; p < 0.001), dyslipidemia (χ^2^ = 22.2; p < 0.001), and smoking (χ^2^ = 23.1; p < 0.001). When segmenting the analysis by subgroups, risk factors associated with complications varied considerably. In the venous procedures group, age (U = 2,174; p < 0.001), DM2 (χ^2^ = 65.01; p < 0.001) and CKD (χ^2^ = 64.78; p < 0.001) were the only risk factors significantly associated with complications.

For the vascular access group, dyslipidemia (χ^2^ = 5.61; p = 0.018), smoking (χ^2^ = 9.09; p = 0.003), and weight (U = 57.0; p = 0.015) were the most relevant predictors. In the arterial procedures group, CKD (χ^2^ = 15.56; p < 0.001) and procedure time (U = 1.956; p = 0.046) were significantly associated with postoperative complications.

The surgical procedure type (χ^2^ = 69.6; p < 0.001) and anesthesia type (χ^2^ = 5.88; p = 0.118) were only assessed in the total sample, with the former being significantly associated with complications. The complete results of the bivariate analysis, including the p-values ​​for each subgroup, are shown in Table 2.

**Table 2:** Association between clinical variables and postoperative complications by subgroups undergoing vascular surgery procedures between 2021 and 2024 at a teaching hospital in western Paraná, Brazil.

**Variable**	**Total sample**	**Venous group**	**Vascular group**	**Arterial group**
Age	**< 0.001**	**< 0.001**	0.09	0.08
Weight	0.18	0.70	**0.01**	0.73
Race	0.45	0.50	1.00	0.38
Sex	0.31	0.31	0.78	0.25
SAH diagnosis	**< 0.001**	0.96	0.63	0.31
DM2 diagnosis	**< 0.001**	**< 0.001**	0.23	0.74
CKD diagnosis	**< 0.001**	**< 0.001**	0.90	**< 0.001**
CAD diagnosis	0.29	0.22	0.69	0.29
Dyslipidemia diagnosis	**< 0.001**	0.07	**0.01**	0.93
History of thrombotic events.	0.12	0.29	0.57	0.31
Chronic use of anticoagulants/antiplatelet agents	0.05	0.95	1.00	0.61
Smoking	**< 0.001**	0.81	**0.003**	0.07
Procedure time (min)	**< 0.001**	0.22	0.25	**0.04**

SAH = systemic arterial hypertension; DM2 = type 2 diabetes mellitus; CKD = chronic kidney disease; CAD = chronic artery disease. Source: research data.

A predictive model was constructed using multivariate binary logistic regression analysis to identify independent factors associated with postoperative complications in the total sample (n = 510). The variables retained in the final model were age, DM2, CKD, dyslipidemia, smoking, chronic use of anticoagulants or antiplatelet agents, procedure duration, and procedure type. In the final model (Table 3), each 1-year increase in age increased the risk by 3.3%, confirming age as an independent risk factor.^2^ CKD was associated with a 9.94-fold increase in the risk of complications, being one of the strongest predictors,^13^ followed by smoking and age. Chronic use of anticoagulants or antiplatelet agents had a significant protective effect. There was a modest association between procedure duration and increased risk. Regarding procedure type, using the venous group as a reference, the vascular access group had a lower risk of complications, while there was no significant association in the arterial group.

**Table 3:** Multivariate logistic regression for postoperative complications in 510 patients undergoing vascular surgery procedures between 2021 and 2024 at a teaching hospital in western Paraná, Brazil.

**Variable**	**Estimates**	**p (< 0.05)**	**OR**	**95% CI**
Age (years)	0.03	0.006	1.03	1.00-1.05
SAH (yes vs no)	-0.32	0.373	0.72	0.35-1.47
DM2 (yes vs no)	0.47	0.181	1.60	0.80-3.23
CKD (yes vs no)	2.29	< 0.001	9.94	4.06-24.3
Dyslipidemia (yes vs no)	0.16	0.681	1.17	0.54-2.55
Smoking (yes vs no)	1.19	0.001	3.29	1.61-6.70
Procedure duration	0.004	0.038	1.00	1.00-1.01
Chronic use of anticoagulants or antiplatelet drugs (yes vs no)	-0.92	0.036	0.39	0.16-0.94
**Surgery type (vs venous procedures)**				
Vascular access procedure	-2.73	<0.001	0.06	0.01-0.23
Arterial procedures	0.40	0.338	1.49	0.65-3.40

DM2 = type 2 diabetes mellitus; SAH = systemic arterial hypertension; CKD = chronic kidney disease; OR = odds ratio; CI 95% = 95% confidence interval. Source: research data.

Risk was calculated based on a multivariate logistic regression model. Each coefficient estimated in the model represents the independent contribution of a predictor variable to the risk of complications. To transform these coefficients (log odds) into clinical probability, the logistic function P(complication) = 1 / (1 + e^–Xβ) was used, where Xβ corresponds to the sum of the intercept and the products between the model coefficients and the values ​​of the variables. Thus, individual risk in relation to the total study population is calculated through the equation:

Probability of complication = 1 / {1 + exp – [–4.64403 + (0.03254 × age) + (0.47558 ​​× DM2) + (2.29688 × CKD) + (1.19200 × smoking) + (0.00494 × procedure duration in minutes) - (0.92193 × anticoagulants/antiplatelet agents) + subgroup coefficient]}.

The model’s performance was evaluated by the area under the receiver operating characteristic curve (AUC). The AUC quantifies the model’s discriminative capacity, ie, how well it separates patients with complications from those without them. The obtained value (Table 4) indicated good model performance (AUC = 0.806; values ​​between 0.8-0.9 are considered good).^36^ The optimal cut-off point identified by the Youden index was 0.181, with a sensitivity of 68.9% and a specificity of 83.3%.

**Table 4:** Discriminative performance of predictive models in different vascular surgery subgroups.

**Model**	**n**	**AUC (95% CI)**	**Cut-off (Youden)**	**Sensitivity**	**Specificity**	**Accuracy**
Total sample	510	0.806 (0.750-0.863)	0.181	68.9%	83.3%	80.8%
Venous group	292	0.803 (0.716-0.891)	0.229	51.5%	96.1%	91.1%
Vascular group	72	0.848 (0.702-0.993)	0.051	100.0%	68.7%	70.8%
Arterial group	146	0.686 (0.588-0.784)	0.320	63.5%	74.5%	70.6%

AUC = area under the receiver operating characteristic curve. Source: research data.

Additionally, specific models were constructed for venous access, vascular access, and arterial procedure subgroups. In the venous procedure group, CKD (OR 15.30; p = 0.007) and DM2 (OR 13.54; p < 0.001) were the main predictors, while age and dyslipidemia were not significant. The model showed satisfactory performance, with AUC = 0.803. The probability of complication in the venous subgroup was estimated by P = 1 / {1 + exp – [-4.0895 + (0.0255 × age) + (2.6058 × DM2) + (2.7281 × CKD) – (0.3466 × dyslipidemia)]}.

In the vascular access group, a trend towards association with smoking was observed (OR 9.57; p = 0.081), although it was not significant. Weight and dyslipidemia were not associated with the outcome. The model demonstrated good discriminatory capacity, with AUC = 0.848. The probability of complication in the vascular access subgroup was estimated by P = 1 / {1 + exp – [5.753 - (0.137 × weight) + (2.259 × smoking) + (0.509 × dyslipidemia)]}.

In the arterial subgroup, CKD was the only predictor significantly associated with complications (OR 6.50; p < 0.001), while procedure duration showed a borderline trend (OR 1.36; p = 0.051). The AUC of the arterial model was 0.686. The probability of complications in the arterial subgroup was estimated by P = 1 /{1 + exp – [-1.762 + (1.871 × CKD) + (0.310 × duration)]}.

## DISCUSSION

The rate of postoperative complications was 17.6%, which is consistent with the national and international literature (ranging from 15% to 30%), depending on the complexity of the procedures and the patient risk profile.^8^ This incidence reinforces the clinical relevance of the problem and the need for tools that help identify higher-risk patients. Preoperative risk assessment is crucial for patient safety, enabling preventive strategies and optimized care.^37^

The predictive model for the total sample identified 8 independent variables associated with the occurrence of postoperative complications: age, DM2, CKD, dyslipidemia, smoking, chronic use of anticoagulants or antiplatelet agents, procedure duration, and procedure type. Overall performance was considered satisfactory (AUC = 0.806), demonstrating adequate discriminatory capacity to differentiate patients at real risk of complications from those with favorable outcomes.^36^

Due to the inherent clinical heterogeneity of vascular surgery types, stratified analysis revealed distinct behaviors among the subgroups. The AUC for the venous access, vascular access, and arterial groups were 0.803, 0.848, and 0.686, respectively, demonstrating good performance of the specific models and reinforcing the importance of segmentation by procedure type. Among the variables, type 2 DM2, CKD, age, and procedure time were the main predictors of complications in the different groups, while anticoagulant use had a protective effect.

Advanced age was an independent predictor of complications in the total sample (p < 0.001) and the venous group (p < 0.001), with the risk increased by 3.3% for each additional year (OR 1.03; p = 0.006). In the vascular (p = 0.09) and arterial (p = 0.08) access groups, there was a trend towards association, although it was not significant. The literature widely confirms age as a risk factor for adverse outcomes in vascular surgery.^38,39^ Older patients have a higher prevalence of comorbidities, lower physiological reserve, and greater frailty, making them more susceptible to complications.^7,38,40^ Managing this population requires thorough preoperative assessment and optimized strategies to mitigate the risks associated with advanced age.

The Brazilian Society of Cardiology’s 2024 Perioperative Cardiovascular Assessment Guidelines^37^ also highlights the importance of age as a risk factor, although, in isolation, it may have low predictive power. However, when combined with other comorbidities, age becomes a robust predictor, as observed in the model.

Among the comorbidities, DM2 was strongly associated with complications (p < 0.001), especially in the venous subgroup, where it was the main risk determinant. This association reflects the impact of DM2 on micro- and macrovasculature, as well as changes in inflammatory and immunological response.^41^ Diabetic patients present with more diffuse and calcified peripheral arterial disease, as well as chronic complications such as neuropathy and nephropathy, which complicate perioperative management and increase the risk of infections, poor wound healing, and cardiovascular events. Recent evidence confirms that DM accelerates the progression of peripheral arterial disease, increasing the likelihood of amputations and cardiovascular mortality.^42^ Thus, multidisciplinary treatment is essential.

CKD was one of the strongest predictors, both in the overall analysis and in the arterial and venous subgroups (p < 0.001), which is consistent with studies that describe it as an independent factor of morbidity and mortality in vascular surgery.^43^ Patients with CKD have a high burden of cardiovascular comorbidities and reduced survival, which are proportional to the severity of renal dysfunction. Studies have shown that CKD and a decrease in the estimated glomerular filtration rate are associated with longer hospital stays, increased postoperative complications, and a need for more intensive perioperative management, especially in complex vascular procedures such as abdominal aortic aneurysm repair, which explains its strong interaction with the arterial subgroup.^44^ Even in its early stages, renal dysfunction requires individualized management, with adjustment to medication dosages and rigorous monitoring of renal function, reinforcing the need for heightened attention in surgical planning.^45^

Smoking was significantly associated with complications in both the total sample (p < 0.001) and the vascular access subgroup (p = 0.03). Smoking is associated with increased perioperative morbidity and mortality, including delayed wound healing, coagulation disorders, and cardiovascular and pulmonary complications. Smokers have an increased risk of infections and other adverse postoperative outcomes.^46^ Smoking cessation in the preoperative period has been shown to significantly reduce these risks, especially when combined with structured interventions that include counseling, pharmacotherapy, and nicotine replacement.^47^ Therefore, the perioperative period should be viewed as a strategic opportunity to encourage behavioral change and optimize clinical outcomes.

The risk of postoperative complications differed among the surgical subgroups. Although complications were more frequent in the arterial procedure group, the association did not remain significant after multivariate adjustment (p = 0.338; OR 1.49), suggesting that the higher risk observed in this group was more related to patient clinical profile than surgery type. However, vascular access had a protective effect against complications (p < 0.001; OR 0.06), consistent with its elective nature and lower technical complexity. When analyzed in isolation, only CKD and procedure time remained significantly associated with the outcome in the arterial subgroup, which could be attributed to clinical homogeneity among these patients and a consequent reduction in statistical power to detect other associations.^39^

Dyslipidemia was significantly associated with complications, especially in the vascular access group (p = 0.01). The literature recognizes that dyslipidemia contributes to the progression of atherosclerotic disease and worse vascular outcomes, although, in the adjusted models, its significance may be attenuated by confounding with other variables.^27,37^ Therefore, its clinical impact can be interpreted as a reflection of the complex interdependence between the multiple risk predictors in vascular patients.

However, hypertension and chronic artery disease did not maintain independent significance in the models adjusted for subgroups (p > 0.05 in all cases). Although they are recognized as cardiovascular risk factors, their effects may have been masked by stronger predictors, such as age, CKD, and DM2. This phenomenon has been previously demonstrated in models such as the New Zealand Vascular Surgical Risk Tool and the Vascular Study Group of New England Cardiac Risk Index, in which traditional variables lose statistical significance after adjustment, without diminishing their clinical relevance.^28^ Thus, a lack of statistical significance does not imply the absence of an effect, but reflects the complexity of the interaction between multiple factors in populations with vascular disease.^48^

Procedure time was also significantly associated with complications (p < 0.001 in the total sample and p = 0.04 in arterial surgeries). Longer procedures increase anesthetic exposure and tissue manipulation, increasing the risk of bleeding, infection, and organ injury. A systematic review and meta-analysis involving different surgical specialties found that procedure time is directly associated with increased morbidity, with an almost doubled risk of complications in surgeries lasting > 2 hours, and that for every additional 30 minutes of procedure time, the risk of complications increases up to 14%,^48^ reinforcing the importance of optimizing intraoperative technical and logistical aspects.

Chronic use of anticoagulants or antiplatelet agents had a protective effect in the overall analysis, suggesting that controlled maintenance of these agents can reduce thrombotic events without significantly increasing the risk of bleeding. This result is consistent with studies highlighting the benefits of carefully managing these medications in the perioperative period for patients with high cardiovascular risk.^49^

The AUC of our predictive model was 0.806, which can be considered good in terms of discrimination, according to criteria established for clinical models and described in methodological guidelines.^35,48,50^ This suggests that the model can reliably distinguish patients with and without a risk of complications, an essential parameter for predictive tools applicable in practice.

This finding is consistent with evidence that specific vascular surgery models tend to outperform^28^ generic scores, such as the Revised Cardiac Risk Index. The New Zealand Vascular Surgical Risk Tool has shown better accuracy in predicting perioperative mortality, while the Vascular Study Group of New England Cardiac Risk Index showed a greater ability to identify cardiac complications than the Revised Cardiac Risk Index.^27^ This reinforces the idea that models focused on the vascular profile better capture risk determinants in this population.

Determining the optimal cut-off point using the Youden index is a well-established practice for balancing sensitivity and specificity.^36^ In the present study, a sensitivity of 68.9% and a specificity of 83.3% were determined with this criterion, demonstrating an adequate balance and good ability to identify patients who will actually present complications, while reducing the risk of false positives and, consequently, unnecessary interventions.

Nevertheless, discriminative performance varied in the subgroup analysis. There was high specificity (96.1%) in the venous group, indicating excellent ability to exclude complications in low-risk patients. Sensitivity (68.7%) and specificity (70.8%) were balanced in the vascular access group. Moderate sensitivity (63.5%) was found for the arterial access group, demonstrating satisfactory overall performance (AUC = 0.686), which is consistent with the greater clinical and technical complexity of these patients. These findings reinforce the validity of the model for different surgical contexts and its potential applicability as a personalized risk stratification tool.

Risk stratification into bands expands the model’s practical utility. This approach facilitates not only interpretation by clinicians but also communication with patients and families, a fundamental aspect in the shared decision-making process. Risk models, such as that proposed in this study, can contribute to more individualized clinical decisions, enabling early identification of patients who will benefit from preventive measures and intensive monitoring. Methodological reviews point out that well-calibrated, validated predictive tools can reduce adverse events and optimize resources in the perioperative setting.^50^

This study involves limitations inherent to its retrospective design and single-center nature. Although the total sample size was adequate and the inclusion and exclusion criteria were rigorous, the division into subgroups reduced the sample size for each category, limiting the statistical power to detect additional associations within each procedure type. Thus, caution should be used when generalizing the results to other populations and surgical contexts. Furthermore, the lack of potentially relevant variables, such as nutritional parameters and inflammatory markers, may also have influenced the predictive performance of the model. External validation in different cohorts is necessary to confirm the tool’s applicability, and the development of a digital interface for the risk calculator would facilitate clinical use, making it a practical tool for risk stratification in vascular surgery.

## CONCLUSIONS

We developed a predictive model capable of estimating postoperative complications in vascular surgery, which achieved good accuracy for the total sample (AUC = 0.806) and consistency between the venous procedure (AUC = 0.803), vascular access (AUC = 0.848), and arterial access (AUC = 0.686) subgroups. In the total sample, advanced age, DM2, CKD, procedure time, and smoking were independent predictors of complications, while chronic use of anticoagulants or antiplatelet agents had a protective effect.

In specific models, DM2 and CKD were observed as the main risk determinants in the venous subgroup, while smoking showed a trend towards association in the vascular access group. In the arterial subgroup, CKD remained the most important factor, with procedure time showing a borderline trend. The model, which has practical applicability for risk stratification and perioperative planning, can guide preventive measures and optimize health care resources. Nevertheless, it requires external validation for confirmation and improvement.

## DISPONIBILIDADE DE DADOS

Os dados que sustentam este estudo estão disponíveis mediante solicitação à autora correspondente, J.P., devido a restrições éticas e de privacidade.
