# Supplementary material for: Risk of complications in vascular surgery: development of a clinical predictive model
Source: J Vasc Bras. 2026 Apr 10;25:e20250181. doi: 10.1590/1677-5449.202501812 (PMC13078178; doi:10.1590/1677-5449.202501812)
Supplement: Translation

# Translation: Risco de complicações em cirurgia vascular: desenvolvimento de modelo clínico preditivo

**DOI:** 10.1590/1677-5449.202501811

**Authors:** Juliana Peres, Jeferson Freitas Toregeani, Ana Julia Vendrametto

**Affiliations:** 1 Centro Universitário Fundação Assis Gurgacz – FAG, Cascavel, PR, Brasil.; 2 Universidade Estadual do Oeste do Paraná – UNIOESTE, Cascavel, PR, Brasil.

**Keywords:** cirurgia vascular, complicações pós-operatórias, avaliação de risco

## Abstract

**Contexto:**

Complicações pós-operatórias em cirurgia vascular estão associadas a elevada morbimortalidade e custos hospitalares, exigindo ferramentas preditivas confiáveis para estratificação de risco.

**Objetivos:**

Desenvolver e validar um modelo clínico para estimar o risco de complicações pós-operatórias em cirurgia vascular.

**Métodos:**

Estudo retrospectivo com 510 doentes submetidos a cirurgias vasculares entre 2021 e 2024, divididos em subgrupos arteriais, venosos e de acesso vascular. Variáveis clínicas e cirúrgicas foram analisadas por regressão logística multivariada, e o desempenho avaliado por meio da curva ROC.

**Resultados:**

A taxa global de complicações foi de 17,6%, sendo maior nos procedimentos arteriais (35,6%) do que nos venosos (11,3%) e de acesso vascular (6,9%). Na amostra total, idade (*odds ratio* [OR] 1,03; p = 0,006), injúria renal crônica (OR 9,94; p < 0,001), tabagismo (OR 3,29; p = 0,001) e tempo de procedimento (p = 0,038) foram preditores independentes, enquanto o uso contínuo de anticoagulantes teve efeito protetor (OR 0,39; p = 0,036). Nos modelos específicos, diabetes melito tipo 2 (OR 13,54; p < 0,001) e injúria renal crônica (OR 15,30; p = 0,007) foram determinantes no grupo venoso; o tabagismo associou-se ao risco no grupo de acesso vascular (OR 9,57; p = 0,081); e a injúria renal crônica manteve significância no grupo arterial (OR 6,50; p < 0,001). O modelo demonstrou bom desempenho discriminativo (área sob a curva ROC [AUC] total = 0,806).

**Conclusões:**

O modelo proposto mostrou boa acurácia e aplicabilidade clínica, permitindo estratificação de risco individualizada em diferentes contextos da cirurgia vascular. A validação externa é necessária para confirmar sua utilidade.

## INTRODUÇÃO

A cirurgia vascular representa um campo essencial e em constante evolução da medicina, abordando uma vasta gama de doenças que afetam o sistema circulatório, desde doenças arteriais obstrutivas periféricas até a correção de aneurismas complexos e a revascularização de territórios nobres, como as artérias carótidas^1,2^. Nas últimas décadas, os avanços nas técnicas cirúrgicas, tanto abertas quanto endovasculares, e o aprimoramento do manejo perioperatório têm permitido o tratamento de pacientes cada vez mais complexos e com múltiplas comorbidades^3,4^. No entanto, apesar desses progressos, as complicações pós-operatórias continuam a ser um desafio significativo, com um impacto profundo na morbidade, mortalidade e qualidade de vida dos pacientes^5-7^.

Estudos multicêntricos e registros de grande escala, como o National Vascular Registry, do Reino Unido, demonstram que a incidência de complicações em cirurgia vascular permanece elevada, com taxas que podem variar de 15 a 30%, dependendo do tipo de procedimento, do caráter de urgência da cirurgia e das condições clínicas basais do paciente^8,9^. Complicações como infecção do sítio cirúrgico, eventos cardiovasculares maiores (infarto agudo do miocárdio, acidente vascular cerebral), insuficiência renal aguda, complicações respiratórias e necessidade de reintervenção são frequentemente relatadas, mesmo em centros de excelência^10-14^. Em particular, a lesão miocárdica após cirurgia não cardíaca tem sido identificada como uma complicação comum e com forte associação com o aumento da mortalidade em curto e longo prazo^7,15^.

O perfil dos pacientes submetidos a cirurgia vascular é caracterizado por uma alta prevalência de fatores de risco cardiovascular, como idade avançada, diabetes melito, hipertensão arterial sistêmica, doença renal crônica e tabagismo^13,16^. Essas comorbidades não apenas aumentam a complexidade do manejo clínico, mas também são preditores independentes de desfechos adversos^17,18^. O impacto dessas condições preexistentes no prognóstico é amplamente documentado, com estudos demonstrando que pacientes com múltiplas comorbidades apresentam maior risco de complicações infecciosas, respiratórias e cardiovasculares, além de maior tempo de internação hospitalar e custos associados^19,20^.

Diante desse cenário, a estratificação de risco individualizada torna-se uma ferramenta fundamental para a tomada de decisão clínica, o planejamento cirúrgico e a otimização do cuidado perioperatório^12^. Diversos escores de risco foram desenvolvidos e aplicados em cirurgia geral e cardíaca, como o Revised Cardiac Risk Index (RCRI)^21^ e o escore da American Society of Anesthesiologists^22^. No entanto, a aplicabilidade e a acurácia desses modelos em coortes de pacientes vasculares são frequentemente limitadas, podendo subestimar o risco em populações de alta complexidade^23-26^.

Como resposta a essa lacuna, ferramentas específicas para a cirurgia vascular têm sido propostas, como o Vascular Study Group of New England Cardiac Risk Index (VSG-CRI)^27^ e o New Zealand Vascular Surgical Risk Tool (NZRISK-VASC)^28^, que demonstraram melhor desempenho em populações específicas^29,30^. Mais recentemente, modelos baseados em aprendizado de máquina e inteligência artificial têm surgido como alternativas promissoras, oferecendo predição dinâmica e personalizada com base em um grande volume de dados^31,32^. Contudo, muitas dessas ferramentas ainda carecem de validação externa ampla ou não são facilmente acessíveis na prática clínica diária.

Portanto, o desenvolvimento de uma calculadora de risco que seja prática, acessível, validada e especificamente desenhada para a população de pacientes submetidos à cirurgia vascular em nosso meio continua a ser uma necessidade premente. O objetivo do presente trabalho é propor a criação de uma ferramenta leve, baseada em preditores independentes de complicações pós-operatórias, que seja clínica e imediatamente aplicável. Tal iniciativa tem potencial de contribuir para a redução de complicações, a melhoria da qualidade assistencial e a segurança do paciente no contexto da cirurgia vascular^28,33,34^.

## MÉTODOS

### Tipo e local de estudo

Este estudo observacional retrospectivo foi aprovado pelo Comitê de Ética em Pesquisa com Seres Humanos do Centro Universitário Fundação Assis Gurgacz (FAG), sob o Certificado de Apresentação de Apreciação Ética número 83271224.7.0000.5219, e número de parecer consubstanciado 7.149.647. Conduzido ao longo de 1 ano, objetivou analisar prontuários de doentes submetidos a cirurgias vasculares em um hospital da região Oeste do Paraná. Considerando sua natureza retrospectiva e a ampla população envolvida no estudo, houve dispensa do Termo de Consentimento Livre e Esclarecido, conforme aprovação ética.

### População do estudo

Este estudo utilizou o método de seleção consecutiva, incluindo todos os doentes submetidos a procedimentos de cirurgia vascular no período de 2021 a 2024, em um hospital escola no Oeste do Paraná. Foram excluídos do estudo sujeitos com idade inferior a 18 anos e aqueles que não possuíam dados pré ou pós-operatórios essenciais para a determinação do desfecho, definido como a ocorrência de complicação pós-operatória.

Para minimizar o risco de seleção, os critérios de inclusão e exclusão foram padronizados e apresentados na Figura 1, garantindo a representatividade da amostra sem interferências subjetivas. As exclusões foram limitadas a casos que poderiam comprometer a análise da pesquisa de complicação, evitando distorções nos resultados. A análise por regressão logística multivariada permitiu o controle de fatores de confusão, tornando a avaliação mais precisa e confiável.

**Figura 1:** Fluxograma da seleção de pacientes.

No total, foram selecionados 602 indivíduos, dos quais 92 foram excluídos. Destes, 51 não possuíam registros pós-operatórios suficientes para indicar se houve complicação, 12 perderam o seguimento clínico após a cirurgia no hospital, e 29 apresentavam registros incompletos quanto à análise epidemiológica.

Para garantir um poder estatístico acordante, calculou-se o tamanho amostral mínimo de 142 participantes, com base em uma fórmula para populações finitas apresentado na Equação 1, considerando incidência de complicações pós cirúrgicas = 15%^8,9,11,21^, n = 510, IC95%, erro = 5%. Entretanto, optou-se por incluir todos os 510 casos elegíveis para aumentar a precisão das estimativas.

Equação 1:


$$\begin{array}{c} \text{n =}\left[\text{510 x 0,15 x}\left(\text{1 - 0,15}\right)\text{x}\left(\text{1,96}\right)\text{²}\right]\text{/}\\ \;\left\{\begin{array}{c} \left[\left(\text{510 - 1}\right)\text{x}\left(\text{0,05}\right)\text{²}\right]\text{+}\\ \left[\text{0,15 x}\left(\text{1 - 0,15}\right)\text{x}\left(\text{1,96}\right)\text{²}\right] \end{array}\right\} \end{array}$$ (1)

### Variáveis analisadas

As variáveis coletadas por meio dos prontuários eletrônicos foram: sexo, idade, peso, etnia, comorbidades preexistentes, como hipertensão arterial sistêmica (HAS), diabetes melito tipo 2 (DM2), doença arterial coronariana (DAC), injúria renal crônica (IRC), dislipidemia, histórico de trombose e tabagismo, além do tipo de procedimento vascular, tempo de procedimento, tipo de anestesia realizada e a ocorrência de complicações pós-operatórias.

### Desfecho principal

O desfecho principal foi a ocorrência de complicações pós-operatórias em pacientes submetidos a cirurgias vasculares. Foram consideradas complicações eventos como necessidade de transfusão sanguínea, infecção de ferida operatória, reintervenção cirúrgica, eventos trombóticos, complicações cardiovasculares e óbito no período pós-operatório imediato. Adicionalmente, buscou-se desenvolver um modelo preditivo de risco, baseado em variáveis clínicas, demográficas e cirúrgicas, com análise estratificada por subgrupos de procedimentos arteriais, venosos e de acesso vascular, a fim de permitir estratificação individualizada do risco e identificação de fatores específicos associados a cada tipo de cirurgia.

### Análise de dados

Os dados coletados foram sintetizados e organizados em uma planilha de Excel®, e analisados na forma descritiva e inferencial. As variáveis ​​contínuas (idade, peso e tempo de procedimento) foram apresentadas como média ± desvio-padrão (DP) para distribuições normais, e como mediana e intervalo interquartil para distribuições não normais. A normalidade foi verificada pelo teste de Shapiro-Wilk. As variáveis ​​categóricas foram expressas como frequências absolutas e percentuais. As comparações entre grupos (doentes com e sem complicações pós-operatórias após cirurgia vascular) para variáveis ​​contínuas foram realizadas utilizando o teste de Mann-Whitney (não paramétrico), em análise por casos completos (*listwise*), em razão da ausência de distribuição normal. As variáveis categóricas foram comparadas utilizando o teste qui-quadrado ou o teste exato de Fisher, quando apropriado. Para identificar fatores associados à ocorrência de complicações pós-operatórias, empregou-se análise de regressão logística binomial, utilizando o método *backward stepwise* e a estatística de Wald. Foram apresentados os valores de *odds ratio* (OR) e respectivos intervalos de confiança de 95% para quantificar a magnitude das associações. Um valor de *p* < 0,05 foi considerado estatisticamente significativo em todas as análises.

As análises descritivas e bivariadas foram realizadas no GraphPad Prism® (versão 10.3.9) e no Jamovi® (versão 2.4). A regressão logística multivariada foi executada no Jamovi®, módulo de regressão, com extração dos coeficientes (β), OR e medidas de ajuste.

O cálculo do risco individual baseou-se nos resultados da regressão logística binária, em que as probabilidades previstas foram categorizadas em baixo (< 20%), intermediário (20-50%) e alto (> 50%) risco, o que facilita a aplicabilidade clínica do escore. As variáveis candidatas ao modelo foram selecionadas a partir de relevância clínica e associação estatística na análise bivariada. O método *backward stepwise*, com estatística de Wald, definiu o modelo final. A função logística aplicou os coeficientes estimados aos preditores clínicos incluídos. As variáveis contínuas foram inseridas em sua forma original (idade em anos), e as categóricas foram codificadas como dicotômicas (0 = ausência; 1 = presença). A calibração e a discriminação do modelo foram avaliadas pela curva ROC, e o ponto de corte para classificação binária (alto *versus* baixo risco) foi definido pelo índice de Youden^35^ (J = sensibilidade + especificidade - 1), que busca o melhor equilíbrio entre sensibilidade e especificidade. Para fins de apresentação, as probabilidades previstas também foram categorizadas em faixas de risco (baixo, intermediário e alto), segundo recomendações da literatura^23^.

## RESULTADOS

Foram avaliados 602 doentes submetidos a procedimentos vasculares, dos quais 92 foram excluídos (51 sem registro pós-operatório, 12 por perda de seguimento e 29 por dados incompletos), totalizando 510 incluídos na análise com informações completas. Devido à heterogeneidade clínica entre os procedimentos da cirurgia vascular, a análise foi estratificada em três subgrupos: arteriais e doenças ateroscleróticas (n = 146), venosos (n = 292) e acessos vasculares (n = 72). Observou-se predomínio do sexo feminino (67,8%) e da raça branca (81,2%). A idade variou entre 18 e 94 anos, com média de 58,8 ± 15,6 anos e mediana de 60. O peso corporal oscilou entre 39 e 150 kg, com média de 73,1 ± 14,2 kg e mediana de 70,2 kg. As características demográficas e clínicas separadas por subgrupos estão apresentadas na Tabela 1.

**Tabela 1:** Características demográficas e clínicas da amostra de 510 pacientes submetidos a procedimentos de cirurgia vascular no período de 2021 a 2024, em um Hospital Escola no Oeste do Paraná, Brasil.

**Variáveis demográficas**	**Venoso (n = 292)**	**Acesso vascular (n = 72)**	**Arterial (n = 146)**
	n (%) ou média ± DP		
Idade (anos)	52,4 ± 13,8	63,4 ± 15,9	69,2 ± 12,1
Peso (kg)	74,9 ± 14,5	72,0 ± 13,0	70,0 ± 13,5
Sexo feminino	232 (79,4%)	39 (54,2%)	75 (51,4%)
Sexo masculino	60 (20,6%)	33 (45,8%)	71 (48,6%)
Brancos	232 (79,5%)	66 (91,7%)	116 (79,5%)
Negros	2 (0,7%)	1 (1,4%)	1 (0,7%)
Pardos	48 (16,4%)	5 (6,9%)	28 (19,2%)
Amarelos	10 (3,4%)	0	1 (0,7%)
Variáveis clínicas diagnosticadas previamente ao procedimento vascular			
Hipertensão	45 (15,4%)	22 (30,6%)	114 (78,1%)
Diabetes melito tipo 2	22 (7,5%)	5 (6,9%)	70 (47,9%)
Doença arterial crônica	3 (1,0%)	2 (2,8%)	23 (15,8%)
Injúria renal crônica	12 (4,1%)	59 (81,9%)	22 (15,1%)
Dislipidemia	16 (5,5%)	7 (9,7%)	78 (53,4%)
Tabagismo	11 (3,8%)	5 (6,9%)	48 (32,9%)
Uso contínuo de anticoagulantes/antiagregantes	9 (3,1%)	0	56 (38,4%)
Paciente com histórico de evento trombótico prévio	9 (3,1%)	4 (5,6%)	38 (26,0%)

DP = desvio-padrão. Fonte: dados da pesquisa.

O procedimento mais frequente foi a cirurgia de varizes bilaterais (57,3%), seguida pela confecção de fístula arteriovenosa (12,5%) e pela revascularização fêmoro-distal (6,7%). Procedimentos de maior complexidade, como reparo de aneurisma de aorta abdominal roto (1,8%) e endarterectomia de carótida (5,5%), também estiveram presentes, embora em menor proporção. Quanto ao manejo anestésico, a técnica predominante variou significativamente entre os grupos. A raquianestesia associada à sedação foi a mais utilizada no grupo venoso (n = 256; 87,7%) e arterial (n = 43; 29,5%), enquanto a anestesia local associada à sedação foi a principal no grupo de acesso vascular (n = 51; 70,8%). A anestesia geral foi mais prevalente no grupo arterial (n = 95; 65,1%). Em relação ao tempo de procedimento, o grupo arterial demonstrou o maior tempo médio, com média de 164 minutos (DP 68,5 min) e mediana de 150 minutos. Em contraste, os grupos venoso e de acesso vascular apresentaram tempos mais curtos, com médias de 102 minutos (DP 41,0 min) e 84,4 minutos (DP 45,2 min), respectivamente.

A taxa de complicação pós-operatória foi de 17,6% (n = 90) na amostra total, com grande disparidade entre os subgrupos. O grupo arterial apresentou a maior taxa de complicações (n = 52; 35,6%), seguido pelo grupo venoso (n = 33; 11,3%) e pelo grupo de acesso vascular (n = 5; 6,9%). Entre os eventos específicos, a necessidade de reoperação foi mais frequente nos grupos arterial (n = 13; 8,9%) e venoso (n = 14; 4,8%), sendo rara no grupo de acessos vasculares (n = 2; 2,8%). A infecção de ferida operatória ou má cicatrização ocorreu em 5,8% dos pacientes arteriais (n = 14) e 5,8% dos venosos (n = 17). Eventos graves, como trombose pós-operatória (n = 11; 7,5%), perda sanguínea intensa intraoperatória (n = 22; 15,1%) e necessidade de transfusão (n = 23; 15,8%), concentraram-se quase exclusivamente no grupo arterial. A injúria renal aguda também foi mais prevalente no grupo arterial (n = 19; 13,0%), e o óbito hospitalar ocorreu apenas nesse subgrupo (n = 7; 4,8%).

Na análise bivariada entre as variáveis clínicas e a ocorrência de complicações pós-operatórias, na amostra total, a idade foi significativamente maior entre os pacientes com complicações (U = 12.513; p < 0,001), assim como o tempo de procedimento (U = 13.924; p < 0,001). Entre as variáveis categóricas, houve associação com complicações para HAS (χ^2^ = 19,2; p < 0,001), DM2 (χ^2^ = 41,9; p < 0,001), IRC (χ^2^ = 16,7; p < 0,001), dislipidemia (χ^2^ = 22,2; p < 0,001) e tabagismo (χ^2^ = 23,1; p < 0,001). Ao segmentar a análise por subgrupos, os fatores de risco associados à complicação variaram consideravelmente. No grupo de procedimentos venosos, a idade (U = 2.174; p < 0,001), a presença de DM2 (χ^2^ = 65,01; p < 0,001) e de IRC (χ^2^ = 64,78; p < 0,001) foram os únicos fatores de risco que se associaram significativamente à ocorrência de complicações.

Para o grupo de procedimentos de acessos vasculares, a dislipidemia (χ^2^ = 5,61; p = 0,018), o tabagismo (χ^2^ = 9,09; p = 0,003) e o peso (U = 57,0; p = 0,015) foram os preditores mais relevantes. Já no grupo de procedimentos arteriais, a IRC (χ^2^ = 15,56; p < 0,001) e o tempo de procedimento (U = 1.956; p = 0,046) foram as variáveis que apresentaram associação estatisticamente significativa com a complicação pós-operatória.

O tipo de procedimento cirúrgico (χ^2^ = 69,6; p < 0,001) e o tipo de anestesia (χ^2^ = 5,88; p = 0,118) foram avaliados apenas na amostra total, sendo o primeiro significativamente associado à complicação. Os resultados completos da análise bivariada, incluindo os valores de p para cada subgrupo, estão detalhados na Tabela 2.

**Tabela 2:** Associação entre variáveis clínicas e complicações pós-operatórias por subgrupos submetidos a procedimentos de cirurgia vascular no período de 2021 a 2024, em um hospital escola no Oeste do Paraná.

**Variável**	**Amostra total**	**Grupo venoso**	**Grupo de acesso vascular**	**Grupo arterial**
Idade	**< 0,001**	**< 0,001**	0,09	0,08
Peso	0,18	0,70	**0,01**	0,73
Raça	0,45	0,50	1,00	0,38
Sexo	0,31	0,31	0,78	0,25
Paciente com diagnóstico de HAS	**< 0,001**	0,96	0,63	0,31
Paciente com diagnóstico de DM2	**< 0,001**	**< 0,001**	0,23	0,74
Paciente com diagnóstico de IRC	**< 0,001**	**< 0,001**	0,90	**< 0,001**
Paciente com diagnóstico de DAC	0,29	0,22	0,69	0,29
Paciente com diagnóstico de dislipidemia	**< 0,001**	0,07	**0,01**	0,93
Paciente com histórico de evento trombótico prévio	0,12	0,29	0,57	0,31
Uso contínuo de anticoagulantes/antiagregantes	0,05	0,95	1,00	0,61
Tabagismo	**< 0,001**	0,81	**0,003**	0,07
Tempo de procedimento (min)	**< 0,001**	0,22	0,25	**0,04**

HAS = hipertensão arterial sistêmica; DM2 = diabetes melito tipo 2; IRC = injúria renal crônica; DAC = doença arterial crônica.

Fonte: dados da pesquisa.

Através da análise por regressão logística binária multivariada, um modelo preditivo foi construído para identificar os fatores independentes associados às complicações pós-operatórias na amostra total (n = 510). As variáveis mantidas no modelo final foram idade, DM2, IRC, dislipidemia, tabagismo, uso contínuo de anticoagulantes ou antiagregantes, duração do procedimento e tipo de procedimento. No modelo final (Tabela 3), cada aumento de 1 ano na idade elevou o risco em 3,3%, confirmando a idade como um fator de risco independente^2^. A presença de IRC foi associada a um aumento substancial de 9,94 vezes na chance de complicações, consolidando-se como um dos mais fortes preditores^13^, seguido pelo tabagismo e pela idade. O uso contínuo de anticoagulantes ou antiagregantes apresentou um efeito protetor significativo. A duração do procedimento demonstrou uma associação modesta com a elevação do risco. Em relação ao tipo de procedimento, tomando o grupo venoso como referência, observou-se que a categoria de acessos vasculares apresentou menor risco de complicações, enquanto o grupo arterial não demonstrou associação significativa.

**Tabela 3:** Regressão logística multivariada para complicações pós-operatórias em 510 pacientes submetidos a procedimentos de cirurgia vascular no período de 2021 a 2024, em um hospital escola no Oeste do Paraná.

**Variáveis**	**Estimativas**	**p (< 0,05)**	**OR**	**IC95%**
Idade (anos)	0,03	0,006	1,03	1,00-1,05
HAS (sim *versus* não)	-0,32	0,373	0,72	0,35-1,47
DM2 (sim *versus* não)	0,47	0,181	1,60	0,80-3,23
IRC (sim *versus* não)	2,29	< 0,001	9,94	4,06-24,3
Dislipidemia (sim *versus* não)	0,16	0,681	1,17	0,54-2,55
Tabagismo (sim *versus* não)	1,19	0,001	3,29	1,61-6,70
Duração do procedimento	0,004	0,038	1,00	1,00-1,01
Uso de anticoagulantes ou antiagregantes de forma contínua (sim *versus* não)	-0,92	0,036	0,39	0,16-0,94
**Tipo de cirurgias (*versus* procedimentos venosos)**				
Procedimento de acesso vascular	-2,73	<0,001	0,06	0,01-0,23
Procedimentos arteriais	0,40	0,338	1,49	0,65-3,40

DM2 = diabetes melito tipo 2; HAS = hipertensão arterial sistêmica; IRC = injúria renal crônica; OR = *odds ratio*; IC95% = intervalo de confiança de 95%. Fonte: dados da pesquisa.

A construção do cálculo de risco fundamentou-se no modelo de regressão logística multivariada. Cada coeficiente estimado no modelo representa a contribuição independente de uma variável preditora sobre a chance de complicação. Para transformar esses coeficientes (*log odds*) em probabilidade clínica, utilizou-se a função logística P(complicação) = 1 / [1 + e^–Xβ], em que Xβ corresponde à soma do intercepto e dos produtos entre os coeficientes do modelo e os valores das variáveis. Assim, o risco individual em relação à população total do estudo é calculado a partir da equação:

Probabilidade de complicação = 1 / [1 + exp - (-4,64403 + (0,03254 × idade) + (0,47558 × DM2) + (2,29688 × IRC) + (1,19200 × tabagismo) + (0,00494 × duração do procedimento em minutos) - (0,92193 × anticoagulantes/antiagregantes) + coeficiente do subgrupo)]

O desempenho do modelo foi avaliado pela área sob a curva ROC (AUC, *area under the curve).* A AUC quantifica a capacidade discriminativa do modelo, ou seja, o quão bem ele separa pacientes que apresentaram complicações daqueles que não apresentaram. O valor obtido (Tabela 4) indica um bom desempenho do modelo (AUC = 0,806; valores entre 0,8-0,9 são considerados bons)^36^. O ponto de corte ótimo identificado pelo índice de Youden foi 0,181, com sensibilidade de 68,9% e especificidade de 83,3%.

**Tabela 4:** Desempenho discriminativo dos modelos preditivos nos diferentes subgrupos de cirurgia vascular.

**Modelo**	**n**	**AUC (IC95%)**	**Ponto de corte (Youden)**	**Sens.**	**Espec.**	**Acurácia**
Amostra total	510	0,806 (0,750-0,863)	0,181	68,9%	83,3%	80,8%
Grupo venoso	292	0,803 (0,716-0,891)	0,229	51,5%	96,1%	91,1%
Grupo de procedimento para acessos vasculares	72	0,848 (0,702-0,993)	0,051	100,0%	68,7%	70,8%
Grupo arterial	146	0,686 (0,588-0,784)	0,320	63,5%	74,5%	70,6%

AUC = *area under the curve* (área sob a curva); Sens. = sensibilidade; Espec. = especificidade. Fonte: dados da pesquisa.

Adicionalmente, foram construídos modelos específicos para os subgrupos de procedimentos venosos, de acesso vascular e arteriais. No grupo de procedimentos venosos, IRC (OR 15,30; p = 0,007) e DM2 (OR 13,54; p < 0,001) foram os principais preditores, enquanto idade e dislipidemia não permaneceram significativas. O modelo apresentou desempenho satisfatório, com AUC = 0,803. A probabilidade de complicação no subgrupo venoso foi estimada por P = 1 / [1 + exp - (-4,0895 + (0,0255 × idade) + (2,6058 × DM2) + (2,7281 × IRC) - (0,3466 × dislipidemia))].

No grupo de procedimentos para acessos vasculares, observou-se tendência de associação para tabagismo (OR 9,57; p = 0,081), embora sem significância. Peso e dislipidemia não se associaram ao desfecho. O modelo demonstrou boa capacidade discriminativa, com AUC = 0,848. A probabilidade de complicação no subgrupo de acessos vasculares foi estimada por P = 1 / [1 + exp - (5,753 - (0,137 × peso) + (2,259 × tabagismo) + (0,509 × dislipidemia))].

Já no subgrupo arterial, a IRC foi o único preditor significativamente associado às complicações (OR 6,50; p < 0,001), enquanto a duração do procedimento apresentou tendência *borderline* (OR 1,36; p = 0,051). A AUC do modelo arterial foi 0,686. A probabilidade de complicação no subgrupo arterial foi estimada por P = 1 / [1 + exp - (-1,762 + (1,871 × DRC) + (0,310 × duração))].

## DISCUSSÃO

A taxa de complicações pós-operatórias no estudo foi de 17,6%, um valor consistente com a literatura nacional e internacional, que reporta taxas variando entre 15 e 30%, a depender da complexidade dos procedimentos e do perfil de risco dos pacientes^8^. Essa incidência reforça a relevância clínica do problema e a necessidade de ferramentas que auxiliem na identificação de pacientes de maior risco. A avaliação de risco pré-operatória é crucial para a segurança do paciente, permitindo a implementação de estratégias preventivas e a otimização do cuidado^37^.

O modelo preditivo da amostra total identificou oito variáveis independentes associadas à ocorrência de complicações pós-operatórias: idade, DM2, IRC, dislipidemia, tabagismo, uso contínuo de anticoagulantes ou antiagregantes, duração do procedimento e tipo de procedimento cirúrgico. O desempenho global foi considerado satisfatório (AUC = 0,806), demonstrando adequada capacidade discriminativa para diferenciar pacientes com risco real de complicações daqueles com evolução favorável^36^.

Devido à heterogeneidade clínica inerente às diferentes categorias da cirurgia vascular, a análise estratificada revelou comportamentos distintos entre os subgrupos avaliados. O grupo venoso apresentou AUC = 0,803, o grupo de acessos vasculares, AUC = 0,848, e o grupo arterial, AUC = 0,686, evidenciando boa performance dos modelos específicos e reforçando a importância da segmentação por tipo de procedimento. Entre as variáveis, DM2, IRC, idade e tempo cirúrgico foram os principais preditores de complicação nos diferentes grupos, enquanto o uso de anticoagulantes demonstrou efeito protetor.

A idade avançada foi um preditor independente de complicações na amostra total (p < 0,001) e no grupo venoso (p < 0,001), com aumento de 3,3% no risco a cada ano adicional (OR 1,03; p = 0,006). Nos grupos de acesso vascular (p = 0,09) e arterial (p = 0,08), observou-se tendência de associação, embora sem significância estatística. A literatura confirma amplamente a idade como fator de risco para desfechos adversos em cirurgia vascular^38,39^. Doentes idosos apresentam maior prevalência de comorbidades, menor reserva fisiológica e maior fragilidade, o que os torna mais suscetíveis a complicações^7,38,40^. O manejo dessa população exige avaliação pré-operatória minuciosa e estratégias otimizadas para mitigar os riscos associados à idade avançada.

A Diretriz de Avaliação Cardiovascular Perioperatória da Sociedade Brasileira de Cardiologia – 2024^37^ também ressalta a importância da idade como fator de risco, embora, isoladamente, possa ter baixo poder preditivo. No entanto, quando combinada com outras comorbidades, a idade se torna um preditor robusto, como observado no modelo.

Entre as comorbidades, o DM2 apresentou associação robusta com complicações (p < 0,001), sobretudo no subgrupo venoso, no qual foi o principal determinante de risco. Essa associação reflete o impacto do DM2 sobre a micro e a macrovasculatura, além de alterações na resposta inflamatória e imunológica^41^. Doentes diabéticos apresentam doença arterial periférica mais difusa e calcificada, bem como complicações crônicas, como neuropatia e nefropatia, que dificultam o manejo perioperatório e elevam o risco de infecções, cicatrização deficiente e eventos cardiovasculares. Evidências recentes confirmam que o DM acelera a progressão da doença arterial periférica, aumentando a probabilidade de amputações e de mortalidade cardiovascular^42^. O manejo multidisciplinar é fundamental.

A IRC foi um dos preditores mais potentes, tanto na análise global quanto nos subgrupos arterial e venoso (p < 0,001), o que está em consonância com estudos que a descrevem como fator independente de morbimortalidade em cirurgia vascular^43^. Doentes com IRC apresentam elevada carga de comorbidades cardiovasculares e redução da sobrevida proporcional à gravidade da disfunção renal. Estudos demonstram que a presença de IRC e a diminuição da taxa de filtração glomerular estimada estão associadas a maior tempo de internação hospitalar, aumento de complicações pós-operatórias e necessidade de manejo perioperatório mais intensivo, especialmente em procedimentos vasculares complexos, como a correção de aneurisma de aorta abdominal, o que explica sua forte interação com o subgrupo arterial^44^. Mesmo em estágios iniciais, a disfunção renal demanda manejo individualizado, com ajuste de doses de medicamentos e monitoramento rigoroso da função renal, reforçando a necessidade de atenção redobrada no planejamento cirúrgico^45^.

O tabagismo teve associação significativa com complicações tanto na amostra total (p < 0,001) quanto no subgrupo de acesso vascular (p = 0,03). O hábito de fumar está associado a maior morbimortalidade perioperatória, incluindo atraso na cicatrização, alterações da coagulação e complicações cardiovasculares e pulmonares. Pacientes tabagistas apresentam risco aumentado de infecções e outros desfechos adversos após procedimentos cirúrgicos^46^. A cessação do tabagismo no período pré-operatório tem demonstrado reduzir significativamente esses riscos, especialmente quando combinada com intervenções estruturadas que associam aconselhamento, farmacoterapia e reposição de nicotina^47^. Assim, o período em torno da cirurgia deve ser encarado como uma oportunidade estratégica para estimular a mudança de comportamento e otimizar os resultados clínicos.

Os subgrupos cirúrgicos apresentaram comportamentos distintos em relação ao risco de complicações pós-operatórias. Embora os procedimentos arteriais tenham ocorrido com maior frequência entre os pacientes que evoluíram com complicações, essa associação não se manteve significativa após o ajuste multivariado (p = 0,338; OR 1,49), sugerindo que o maior risco observado nesse grupo está mais relacionado ao perfil clínico dos pacientes do que ao tipo de cirurgia em si. No entanto, o procedimento de acesso vascular apresentou efeito protetor (p < 0,001; OR 0,06), compatível com sua natureza eletiva e menor complexidade técnica. Quando analisado isoladamente, o subgrupo arterial manteve associação estatística com o desfecho apenas para a IRC e o tempo de procedimento, o que pode ser atribuído à homogeneidade clínica entre os pacientes arteriais e à consequente redução do poder estatístico para detectar outras associações^39^.

A dislipidemia mostrou associação significativa com complicações, especialmente no grupo de acesso vascular (p = 0,01). A literatura reconhece que a dislipidemia contribui para a progressão da doença aterosclerótica e para a piora dos desfechos vasculares, ainda que, em modelos ajustados, sua significância possa ser atenuada por confundimento com outras variáveis^27,37^. Portanto, seu impacto clínico pode ser interpretado como reflexo da interdependência complexa entre os múltiplos preditores de risco que compõem o perfil dos pacientes vasculares.

Por outro lado, HAS e DAC não mantiveram significância independente nos modelos ajustados por subgrupos (p > 0,05 em todos os casos). Embora sejam reconhecidos como fatores de risco cardiovascular, seus efeitos podem ter sido mascarados por preditores mais fortes, como idade, IRC e DM2. Esse fenômeno já foi demonstrado em modelos como o NZRISK-VASC e o VSG-CRI, nos quais variáveis tradicionais perdem significância estatística após ajuste, sem que isso diminua sua relevância clínica^28^. Assim, a ausência de significância estatística não implica ausência de efeito, mas reflete a complexidade da interação entre múltiplos fatores em populações vasculares^48^.

O tempo de procedimento cirúrgico também apresentou relação significativa com complicações (p < 0,001 na amostra total e p = 0,04 nas cirurgias arteriais). Procedimentos mais longos aumentam a exposição anestésica e a manipulação tecidual, elevando o risco de sangramento, infecção e injúria orgânica. Uma revisão sistemática e meta-análise envolvendo diferentes especialidades cirúrgicas demonstrou que a duração do procedimento está diretamente associada ao aumento da morbidade, com risco quase duplicado de complicações em cirurgias com duração superior a duas horas, e que a cada 30 minutos adicionais de tempo operatório há incremento de até 14% no risco de complicação^48^, reforçando a importância da otimização técnica e logística intraoperatória.

O uso contínuo de anticoagulantes ou antiagregantes demonstrou efeito protetor na análise global, sugerindo que a manutenção controlada desses agentes pode reduzir eventos trombóticos sem aumento expressivo do risco hemorrágico. Esse resultado está em concordância com estudos que destacam a segurança do manejo criterioso dessas medicações no perioperatório de pacientes com alto risco cardiovascular^49^.

O modelo preditivo desenvolvido apresentou uma AUC de 0,806, considerada boa em termos de discriminação, conforme critérios estabelecidos para modelos clínicos e descritos em diretrizes metodológicas^35,48,50^. Esse desempenho sugere que o modelo é capaz de distinguir de maneira confiável pacientes em risco de complicações daqueles sem risco, parâmetro essencial para ferramentas preditivas aplicáveis na prática.

Esse achado é consistente com evidências de que modelos específicos para cirurgia vascular tendem a superar^28^ escores genéricos, como o RCRI. O NZRISK-VASC demonstrou melhor acurácia na predição de mortalidade perioperatória, enquanto o VSG-CRI mostrou maior capacidade de identificar complicações cardíacas quando comparado ao RCRI^27^. Isso reforça que modelos direcionados ao perfil vascular capturam melhor os determinantes de risco dessa população.

A determinação do ponto de corte ótimo por meio do índice de Youden é uma prática consolidada na literatura para equilibrar sensibilidade e especificidade^36^. No presente estudo, esse critério definiu uma sensibilidade de 68,9% e especificidade de 83,3%, evidenciando equilíbrio adequado e boa capacidade de identificar pacientes que realmente apresentarão complicações, ao mesmo tempo em que reduz o risco de falsos positivos e, consequentemente, de intervenções desnecessárias.

Quando analisado por subgrupos, observou-se variação do desempenho discriminativo. O grupo venoso apresentou alta especificidade (96,1%), o que reflete excelente capacidade de exclusão de complicações em pacientes de baixo risco; o grupo de acessos vasculares manteve equilíbrio entre sensibilidade (68,7%) e especificidade (70,8%); e o grupo arterial, embora com sensibilidade moderada (63,5%), demonstrou desempenho global satisfatório (AUC = 0,686), compatível com a maior complexidade clínica e técnica desses pacientes. Esses achados reforçam a validade do modelo para diferentes contextos cirúrgicos e seu potencial de aplicabilidade como ferramenta de estratificação de risco personalizada.

A estratificação do risco em faixas amplia a utilidade prática do modelo. Essa abordagem facilita não apenas a interpretação pelos clínicos, mas também a comunicação com pacientes e familiares, aspecto fundamental no processo de tomada de decisão compartilhada. Modelos de risco como o proposto podem contribuir para decisões clínicas mais individualizadas, permitindo identificar precocemente pacientes que se beneficiarão de medidas preventivas e de monitorização intensiva. Revisões metodológicas destacam que ferramentas preditivas bem calibradas e validadas têm potencial para reduzir eventos adversos e otimizar recursos no contexto perioperatório^50^.

Este estudo apresenta limitações inerentes ao seu desenho retrospectivo e à sua natureza de centro único. Embora a amostra total seja adequada e os critérios de inclusão e exclusão sejam rigorosos, a divisão em subgrupos reduz o tamanho amostral de cada categoria, o que limita o poder estatístico para detectar associações adicionais dentro de cada tipo de procedimento. Assim, a generalização dos resultados para outras populações e contextos cirúrgicos deve ser interpretada com cautela. Além disso, a ausência de variáveis potencialmente relevantes, como parâmetros nutricionais e marcadores inflamatórios, também pode ter influenciado o desempenho preditivo do modelo. A validação externa em diferentes coortes é necessária para confirmar a aplicabilidade. Além disso, o desenvolvimento de uma interface digital para a calculadora facilitaria a utilização clínica, tornando-a uma ferramenta prática para estratificação de risco em cirurgia vascular.

## CONCLUSÃO

O estudo desenvolveu um modelo preditivo capaz de estimar complicações pós-operatórias em cirurgia vascular considerando a amostra total, com boa acurácia (AUC = 0,806) e consistência entre os subgrupos de procedimentos venosos (AUC = 0,803), de acessos vasculares (AUC = 0,848) e arteriais (AUC = 0,686). Na amostra total, idade avançada, DM2, IRC, tempo de procedimento e tabagismo foram preditores independentes de complicações, enquanto o uso contínuo de anticoagulantes ou antiagregantes demonstrou efeito protetor.

Nos modelos específicos, observou-se que DM2 e IRC foram os principais determinantes de risco no subgrupo venoso, enquanto o tabagismo apresentou tendência de associação no grupo de acessos vasculares. Já no subgrupo arterial, a IRC manteve-se como o fator de maior impacto, com a duração do procedimento mostrando tendência limítrofe. O modelo apresenta aplicabilidade prática para estratificação de risco e planejamento perioperatório, podendo orientar medidas preventivas e otimizar recursos assistenciais. Adicionalmente, necessita de validação externa para confirmação e aprimoramento.

## Data Availability

The data supporting this study are available upon request from the corresponding author, J.P., due to ethical and privacy restrictions.
